# O Legado do Professor Eduardo Sosa

**DOI:** 10.36660/abc.20201080

**Published:** 2020-11-01

**Affiliations:** 1 Universidade de São Paulo Instituto do Coração (Incor) São PauloSP Brasil Instituto do Coração (Incor) – Universidade de São Paulo (USP), São Paulo, SP – Brasil

**Keywords:** Eduardo Sosa, Cardiologia, Doenças Cardiovasculares, Eletrocardiografia, Eletrofisiologia, Técnicas Eletrofisiológicas/tendências, Técnicas Eletrofisiológicas Cardíacas/tendências, Pesquisadores, Docentes/história

Em 20 de junho de 2020, recebemos uma triste notícia: o falecimento do Professor Dr. Eduardo Argentino Sosa, Professor Associado de Cardiologia do Instituto do Coração da Universidade de São Paulo. Essa informação provocou grande consternação em todos que o conheceram.

## Breve História

Eduardo Argentino Sosa nasceu em Corrientes, Argentina, em 25 de janeiro de 1942. Iniciou o curso de medicina na Faculdade de Ciências Médicas da Universidade Nacional do Nordeste, na sua cidade natal; no entanto, devido à situação política que afetava o funcionamento universitário na Argentina de então, transferiu-se para a Faculdade de Ciências Médicas de Córdoba e, posteriormente, para a Faculdade de Ciências Médicas da Universidade Nacional do Litoral, na cidade de Rosário, onde concluiu o curso em abril de 1965.^1^

Seu interesse pela cardiologia surgiu durante o internato, quando ficou fascinado pelos métodos diagnósticos existentes, tais como eletrocardiografia, vetocardiografia, fonomecanocardiografia e cateterismo cardíaco, que se iniciava na época. Daí surgiu o seu primeiro trabalho científico, apresentado na 1ᵃ Jornada Nacional de Cardiologia da Federação Argentina de Cardiologia, na cidade de Carlos Paz, em Córdoba, intitulado “Bloqueo de Rama Izquierda Intermitente. Estudo Fonográfico e Poligráfico”. Logo foi contratado como médico assistente do serviço de cardiologia, e lá conheceu Miguel Barbero Marcial, que se tornaria seu amigo pessoal e mudaria o rumo de sua vida.^1^

No período de 1966 e 1967, ainda em Rosário, viajava semanalmente para Buenos Aires para aprender as técnicas de cateterismo no Hospital Ramos Mejia, no serviço do Professor Blas Moia, e eletrocardiografia no Hospital de Salaberry, no serviço do Professor Marcelo Rosembaum.^1^

Em uma dessas visitas conheceu o professor Demétrio Sodi Pallares, do Instituto Nacional de Cardiologia do México, na época, um dos centros de referência mundial de pesquisa em cardiologia.

Sosa planejava fazer a complementação do seu treinamento no México. Contudo, seu amigo Miguel havia se mudado para São Paulo e o convenceu a vir para o Hospital das Clínicas da Faculdade de Medicina da Universidade de São Paulo (HC-FMUSP). O motivo? Ele percebeu que havia uma verdadeira revolução em andamento no tratamento das doenças cardiovasculares com os avanços da cirurgia cardiovascular, e encontrou no HC-FMUSP um grupo acadêmico excepcional, que trabalhava com entusiasmo e inspiração, sob a coordenação dos Professores Euryclides de Jesus Zerbini e Luiz Venere Décourt.

**Figura 1:**
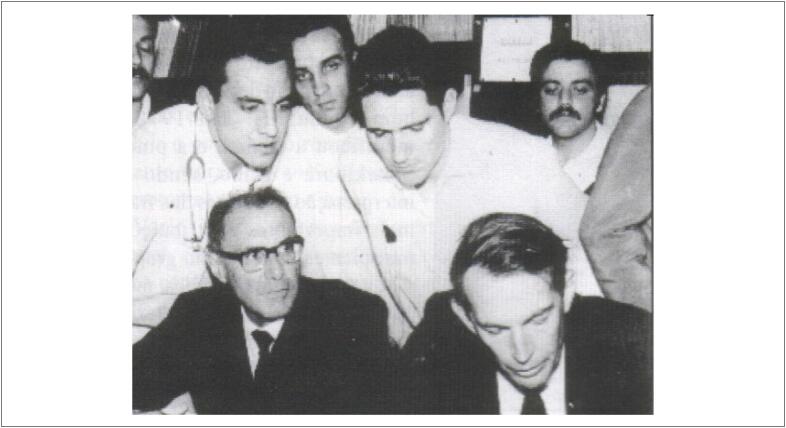
Foto de 1968 documentando a visita do Professor Christiaan Barnard em visita ao HC-FMUSP. Da direita para a esquerda, Professor Christiaan Barnard, Professor Euryclides de Jesus Zerbini, Eduardo Sosa, Noedir Stolf e colaboradores da época.

Sosa chegou em São Paulo no dia 13 de fevereiro de 1968, sendo admitido de imediato no curso do Professor Décourt, um ícone da cardiologia brasileira. Percebendo seu potencial acadêmico, o Professor Giovanni Bellotti, assistente de Décourt, o “adotou” e o tornou um dos colaboradores da equipe que faria os primeiros transplantes de coração na América do Sul (Figura 1). Por sua dedicação, recebeu convite para permanecer definitivamente no Brasil.

Em 1972, teve seu diploma de médico revalidado e, em 1974, foi contratado após concurso público, como médico assistente na Segunda Clínica Médica do Hospital das Clínicas, passando a trabalhar com Giovanni Bellotti no Grupo de Válvulas. Assim, ingressou na Clínica de Cardiologia como responsável pelos leitos daquele grupo.

Sosa sempre teve grande comprometimento com o bem-estar do paciente e também gostava de ensinar, motivando seus residentes e internos com intervenções brilhantes. No entanto, não se sentia completamente realizado, pois queria grandes desafios. Quem está tendo o primeiro contato com Sosa nessa homenagem perceberá que estamos falando da cardiologia brasileira de quase 50 anos atrás. Tempo de poucos procedimentos, poucos medicamentos e muitas doenças, com conhecimentos muito mais limitados do que atualmente.

Seu interesse pela eletrofisiologia surgiu após ter acesso ao trabalho pulicado por Scherlag et al., publicado em 1969, demonstrando a viabilidade do registro do eletrograma do feixe de His em humanos, por acesso vascular.^2^ No ano seguinte, em conjunto com os professores Giovanni Bellotti, João Tranchesi, Radi Macruz e Donaldo Pereira Garcia, conseguiu obter o primeiro registro do eletrograma do feixe de His no HC-FMUSP, e, em 1972, realizaram a primeira tentativa de cirurgia de Wolff-Parkinson-White (WPW).^3^

Um dia, em 1974, Bellotti procurou Sosa e o convocou para estudar eletrofisiologia na síndrome de WPW para sua tese de doutorado. Tiveram então que aprender a analisar traçados eletrofisiológicos. Assim, Sosa foi apresentado a esse novo mundo com inúmeros desafios.

O primeiro foi conversar com o Professor Antonio Paes de Carvalho, Professor Titular do Instituto de Biofísica e Fisiologia Carlos Chagas da Universidade Federal do Rio de Janeiro (UFRJ), grande cientista, estudioso dos mecanismos eletrofisiológicos das arritmias cardíacas em nível experimental e reconhecido internacionalmente.^4^ Com ele, Sosa foi aprender como obter e analisar os eletrogramas intracardíacos.

**Figura 2:**
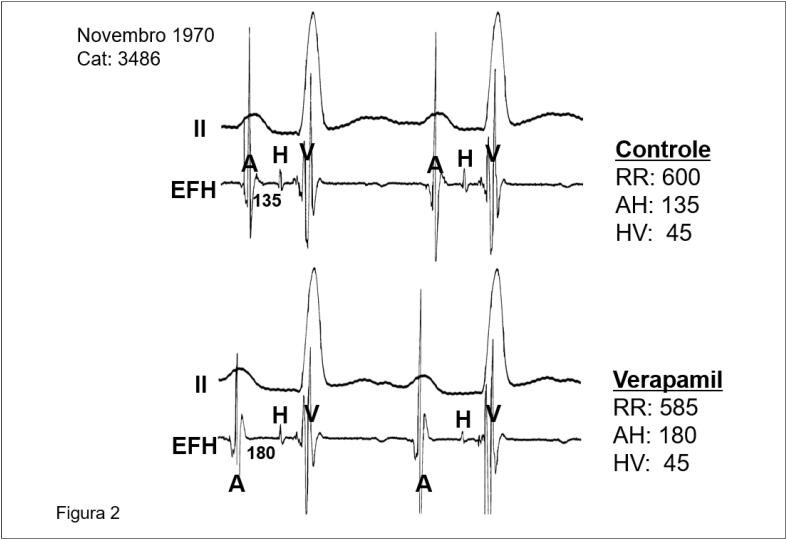
Registro eletrofisiológico obtido no HC-FMUSP em 1970 documentando o efeito da infusão venosa de verapamil nos intervalos básicos do sistema de condução.

Depois de alguns dias fazia o primeiro estudo eletrofisiológico no HC, com Bellotti. O interessante é que os primeiros traçados eram rudimentares, com interferências e deflexões por toda parte. Bellotti perguntava: “Sosa, qual é o H?”; como havia três deflexões, a dúvida não foi solucionada, mas aprenderam que os registros deveriam ter menos interferências para serem confiáveis. Sosa, obstinado pela perfeição, elaborou meios de conseguir registros de alta qualidade para a época (Figura 2).

Em seguida, começaram os estudos dos pacientes para a tese de Bellotti. Eles passavam o dia induzindo taquicardias com estímulos induzidos por eletrodos, analisando traçados e interpretando os achados. Ao final de cada estudo, sentiam uma satisfação intensa pelos achados.

Contudo, a maior surpresa ocorreu no dia da defesa da tese de Bellotti. No final da apresentação a banca examinadora elogiou muito o trabalho inovador e encerrou a arguição em cerca de 20 minutos. Até hoje, essa foi uma das defesas de tese de menor duração da universidade, possivelmente porque a banca pouco ou nada sabia a respeito do assunto.^5^

Em 1975, Bellotti foi nomeado chefe da unidade de terapia intensiva (UTI) de cardiologia, no 6º andar do HC. Nessa altura, José Antonio Frachini Ramires começou a trabalhar com ele como assistente de clínica médica e UTI de cardiologia. “Quando encontrávamos Sosa pelos corredores do hospital, com rolos de traçados eletrofisiológicos nos braços, e caso ele quisesse nos mostrar algum detalhe, deveríamos nos preparar para passar um longo tempo ajudando-o a desdobrá-los, ver os registros e depois dobrá-los novamente. Alguns assistentes da clínica fugiam desses encontros”, lembra Ramires.

Nessa ocasião, Cesar Grupi juntou-se ao grupo, auxiliando-o na realização dos primeiros estudos eletrofisiológicos.^6^^,^^7^ Naquela época, os estudos foram realizados em diferentes condições, como em pacientes com infarto agudo do miocárdio por sugestão do Professor Radi Macruz e avaliação do efeito eletrofisiológico dos fármacos antiarrítmicos, como grandes novidades…^8^^,^^9^

Com a transferência da cardiologia para o Incor, que acabara de ser inaugurado, Sosa acumulou a liderança do grupo de válvulas e o embrião do grupo de arritmias. Por seu pedido, Bellotti e o Professor Fúlvio Pilleggi criaram dois grupos independentes: o de arritmia e o de valvopatias, que assim permanecem até os dias atuais.

O laboratório de eletrofisiologia cardíaca foi estruturado em 1980, tornando-se fundamental para o desenvolvimento do tratamento intervencionista de várias arritmias no Incor. Buscando aprimoramento para os seus procedimentos, Sosa e Miguel visitaram serviços nos EUA, em particular de Kenneth Rosen, em Chicago, e de Mark Josephson, na Filadélfia.

Vale ressaltar que, quando visitaram esses serviços, levavam na bagagem 10 cirurgias de WPW e 10 cirurgias de taquicardia ventricular, o que impressionou os professores locais, pois pouquíssimos serviços americanos realizavam esses procedimentos na época.

**Figura 3:**
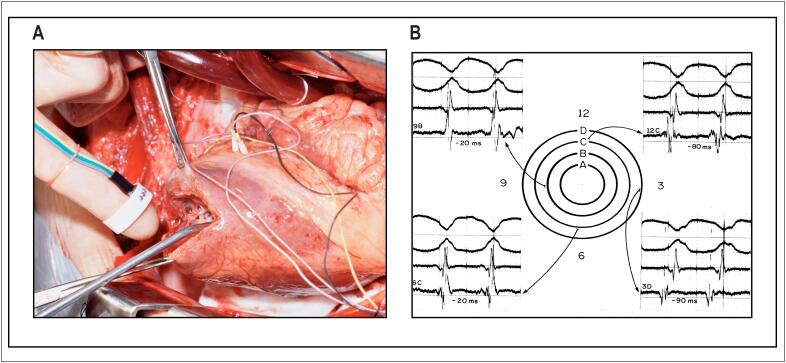
A e B) Mapeamento intraoperatório de um paciente com taquicardia ventricular recorrente secundária à cardiopatia chagásica nos anos 1980. A) O acesso do mapeamento endocárdico era pelo aneurisma apical. Note que o mapeamento ponto a ponto era realizado por eletrodos bipolares anexados em um dispositivo que permitia seu posicionamento direto pelo dedo do cirurgião. B) Os registros eletrofisiológicos documentam a sequência de ativação endocárdica da taquicardia ventricular induzida na cirurgia por estimulação programada.

Notavelmente, Sosa e Miguel mudaram o paradigma do tratamento de arritmias na doença de Chagas, demonstrando que as taquicardias ventriculares sustentadas recorrentes eram circuitos reentrantes relacionados com cicatrizes, e podiam ser reproduzidas com estimulação ventricular programada. Além disso, mais importante, observaram que tais circuitos originavam-se com maior frequência em uma cicatriz localizada na parede inferior, lateral e basal do ventrículo esquerdo, pois, até então, acreditava-se que o aneurisma apical, tão frequentemente em pacientes com doença de Chagas, fosse o foco de tais arritmias^10^ (Figura 3).

Ao longo dos anos 1980 e 1990, o programa de tratamento cirúrgico para taquiarritmias atriais, supraventriculares e ventriculares progrediu intensamente, tornando o Incor um centro de referência no tratamento cirúrgico de taquiarritmias refratárias e de treinamento de eletrofisiologistas e cirurgiões, não somente do Brasil como da América Latina.^10^^–^^15^

Em 1982, Gallagher et al.^16^ publicaram a primeira experiência com ablação por cateter para induzir bloqueio atrioventricular total em pacientes com taquiarritmias supraventriculares intratáveis. Uma vez ciente desse novo avanço, Sosa conseguiu desenvolver conexões entre o desfibrilador e cateteres convencionais, com o auxílio de Adib Jatene.

Em conjunto com Augusto Scalabrini e Silvio Barbosa, fizeram a primeira ablação por cateter com sucesso, utilizando descarga elétrica direta (fulguração) para indução de bloqueio atrioventricular total (BAVT).^17^ Esses procedimentos eram realizados com cateteres eletrodos convencionais, como ocorria nos poucos serviços internacionais que também passaram a realizá-lo. Assim foi desenvolvido o primeiro laboratório estruturado de eletrofisiologia intervencionista do Brasil, fruto de seu espírito inovador.

Durante sua gestão como diretor da unidade de arritmia, ainda nos anos 1980, Sosa estimulou o desenvolvimento da área clínica da estimulação cardíaca artificial, contando com Martino Martinelli como assistente responsável e Silvana d'Ório e Anísio Pedrosa como colaboradores, o que resultou na unidade de arritmias e marca-passo.^18^^,^^19^

Com a ampliação do InCor e aumento do número de salas de atendimento ambulatorial, no início dos anos 1990, Sosa incentivou a criação do ambulatório de síncope acoplado ao laboratório de avaliação autonômica; o ambulatório didático; de fibrilação atrial; de taquicardia ventricular e de genética, e contou com a colaboração de novos assistentes, Denise Hachul e Francisco Darrieux, que desenvolveram em conjunto um programa de extensão universitária em arritmias clínicas.^20^^,^^21^

Retornando aos anos 1970, Ramires recorda que em sua tese de mestrado sobre o tema do bloqueio autonômico em chagásicos, Sosa inspirou o uso de betabloqueadores em pacientes com infarto agudo do miocárdio e insuficiência cardíaca. Foi comprovado seu benefício por meio de avaliação metabólica miocárdica e monitoramento hemodinâmico à beira do leito. A partir de então, foi introduzido o uso de betabloqueadores na rotina terapêutica de ambas as patologias em nossa instituição.^22^^,^^23^

Seu grande interesse acadêmico, no entanto, sempre foi a área de eletrofisiologia intervencionista, na qual trabalhou intensamente com seu assistente, Mauricio Scanavacca. Formaram inúmeros eletrofisiologistas ao longo desses anos, atualmente responsáveis por serviços de arritmia e eletrofisiologia não somente do Brasil, mas também no exterior.^24^^–^^31^

**Figura 4:**
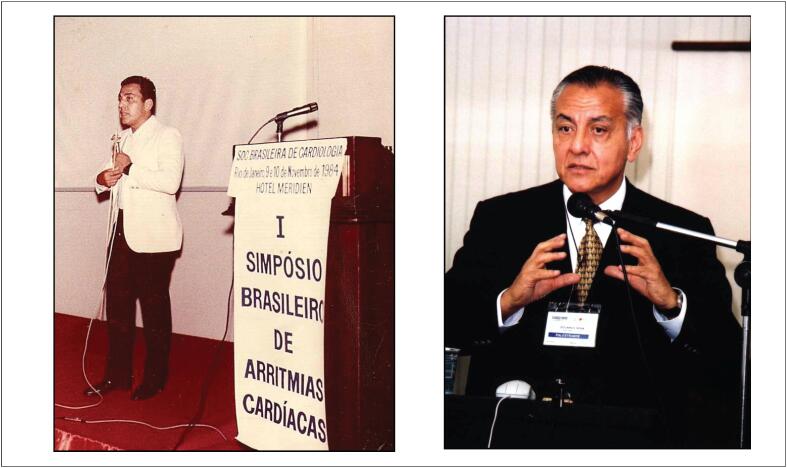
Dois momentos do Dr. Sosa participando das reuniões científicas da especialidade. A) Ainda jovem, na primeira Jornada de Arritmias Cardíacas da SBC no Rio de janeiro, em 1984. B) Já amadurecido, em meados dos anos 2000, em um dos congressos do Departamento de Arritmias Cardíacas da SBC.

Em 1984, junto com os colegas cardiologistas envolvidos no tratamento de pacientes com arritmias cardíacas – em particular, Ivan Maia (no Rio de Janeiro), Adaberto Lorga (em São José do Rio Preto), João Pimenta (no Hospital do Servidor Público Estadual) e Julio Gizzi (no Instituto de Cardiologia Dante Pazzanese de Cardiologia), ambos em São Paulo –, fundaram o Grupo de Estudo de Arritmia, Eletrofisiologia e Estimulação Cardíaca Artificial, embrião do que se tornaria o departamento de eletrofisiologia da Sociedade Brasileira de Cardiologia (SBC) e, posteriormente, a Sociedade Brasileira de Arritmias Cardíacas (SOBRAC) (Figura 4A e B).

Em meados dos anos 1990, o grande desafio enfrentado pela eletrofisiologia era conseguir melhores resultados na ablação da taquicardia ventricular (TV). Em nosso meio, esse desafio parecia maior em pacientes com cardiopatia chagásica.

**Figura 5:**
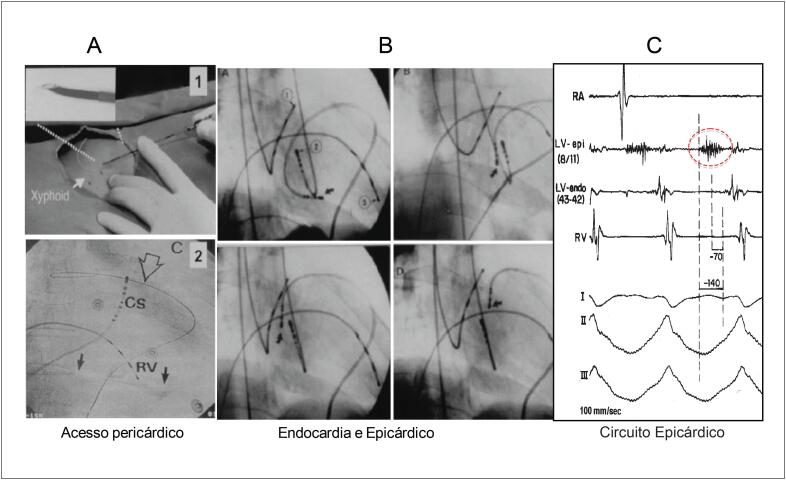
Figuras da Capa do Journal of Cardiovascular Electrophysiology de junho de 1996 divulgando o artigo que descreveu a técnica de acesso percutâneo do espaço pericárdico. A. Acesso pericárdico por punção subxifoide. B. Mapeamento epicárdico com cateter. C. Registros dos eletrogramas bipolares durante taquicardia ventricular em um paciente com doença de Chagas demonstrando a presença de um circuito epicárdico.

Após constatarem a baixa taxa de sucesso das ablações nesses pacientes, Sosa, Mauricio e o anestesista da equipe, João Piccioni, desenvolveram a técnica de mapeamento e ablação epicárdica da taquicardia ventricular e demonstraram que os circuitos eram predominantemente epicárdicos nos pacientes com cardiopatia chagásica^32^^–^^36^ (Figura 5). As observações obtidas pelo grupo, nessa época com a participação de André D'Ávila, alcançaram o cenário internacional, e a técnica passou a ser utilizada em outros tipos de arritmias, além da TV chagásica.^31^^,37^

Desde então, os congressos das sociedades americanas e europeias de arritmia e cardiologia têm sido palco constante de apresentações da técnica de mapeamento e ablação epicárdica, incluindo sessões exclusivamente dedicadas, muitas das quais presididas e moderadas pelos seus idealizadores.

Após a publicação do trabalho com os resultados da nova técnica, inúmeros eletrofisiologistas europeus, latino-americanos e norte-americanos procuraram o Incor para um treinamento na técnica na Unidade de Arritmia e Eletrofisiologia, a fim de implementá-la em seus serviços, como a Cleveland Clinic, Mayo Clinic, Universidade da Califórnia, Stanford, Massachusetts General Hospital e vários Centros de Referência Europeus. Foi um dos momentos culminantes da obra do “insatisfeito” Sosa, que sempre buscava mais.

Não há dúvida de que Sosa criou uma escola com muitos discípulos por todo o Brasil, América Latina e outros continentes. Eduardo Argentino Sosa, argentino de nascimento e brasileiro de coração, tinha visão focada no infinito.

Eduardo Sosa permanecerá para sempre na lembrança de seus amigos, na história do Incor e da cardiologia brasileira, e seu legado será eternizado por seus discípulos.
