# Supplementary material for: O Legado do Professor Eduardo Sosa
Source: Arq Bras Cardiol. 2020 Nov 1;115(5):801–6. [Article in Portuguese] doi: 10.36660/abc.20201080 (PMC8452212; doi:10.36660/abc.20201080)
Supplement: Translation

# Translation: The legacy of Prof. Eduardo Sosa

**Affiliations:** 1 São PauloSP Brazil Instituto do Coração (Incor) – Universidade de São Paulo (USP), São Paulo, SP – Brazil

**Keywords:** Eduardo Sosa, Cardiology, Cardiovascular Diseases, Electrocardiography, Electrophysiology, Electrophysiologic Techniques Cardiac/trends, Research Personnel, Faculty/history

On June 20, 2020, we received some sad news: the death of Professor Dr. Eduardo Argentino Sosa, Associate Professor of Cardiology at Instituto do Coração da Universidade de São Paulo. This information filled everyone who knew him with deep sadness.

## A Short Background

Eduardo Argentino Sosa was born in Corrientes, Argentina, on January 25, 1942. He started a medical course at the School of Medical Sciences of Universidad Nacional del Nordeste, in his hometown, but due to the political situation that affected the university in Argentina at that time, he transferred to the School of Medical Sciences of Córdoba and later to the School of Medical Sciences of Universidad Nacional del Litoral, in the city of Rosário, where he completed studies in April 1965.^1^

His interest in cardiology arose during his internship, when he was fascinated by the existing diagnostic methods, such as electrocardiography, vetocardiography, phono-cardiography and cardiac catheterization, which began at the time. Hence his first scientific study, presented at the 1^st^ National Cardiology Day at the Argentine Federation of Cardiology, in the city of Carlos Paz, in Córdoba, entitled “Intermittent Left Branch Block. Phonographic and Polygraphic Study.” He was soon hired as an assistant physician in the cardiology service, where he met Miguel Barbero Marcial, who later became his personal friend and changed the course of his life.^1^

From 1966 to 1967, still in Rosario, he traveled weekly to Buenos Aires to learn the techniques of catheterization at Hospital Ramos Mejia, at the service of Professor Blas Moia and Electrocardiography at Hospital de Salaberry, at the service of Professor Marcelo Rosembaum.^1^

On one of those visits, he met Professor Demétrio Sodi Pallares, from the National Institute of Cardiology in Mexico, at the time a world reference center in cardiology research.

Sosa planned to further develop his education in Mexico. However, his friend Miguel had moved to São Paulo and convinced him to come to Hospital das Clínicas, School of Medicine of Universidade de São Paulo (HC-FMUSP). The reason? He realized that there was an actual revolution underway in the treatment of cardiovascular diseases, with the advances of cardiovascular surgery and found in HC-FMUSP an exceptional academic group that worked with enthusiasm and inspiration, coordinated by Professors Euryclides de Jesus Zerbini and Luis Venere Décourt.

Sosa arrived in São Paulo on February 13, 1968, and was immediately admitted to the course of Prof. Décourt, an icon of Brazilian cardiology. Noticing his academic potential, Professor Giovanni Bellotti, assistant to Décourt, “adopted” him and made him one of the members of the team that did the first heart transplants in South America. (Figure 1) For his dedication, he was invited to stay permanently in Brazil.

**Figure 1:** Photo from 1968 documenting Prof. Christiaan Barnard visiting Hospital das Clínicas of FMUSP. From right to left, Professor Christiaan Barnard, Professor Euriclides de Jesus Zerbini, Eduardo Sosa, Noedir Stolf and contributors of the time.

In 1972, he had his medical diploma revalidated and, in 1974, he was hired through a public examination as an assistant physician at Segunda Clínica Médica do Hospital das Clínicas and started to work with Giovanni Bellotti in the Valves Group. He was then practicing Cardiology and was responsible for the patients seen in that group.

Sosa has always had a strong commitment to the patients' well-being and also enjoyed teaching and motivating residents and interns with brilliant interventions. However, he did not feel completely fulfilled, as he wanted great challenges. Whoever is having the first contact with Sosa in this tribute will realize that we are talking about Brazilian cardiology of almost 50 years ago. A time of few procedures, few medications and many diseases, with much more limited knowledge than today.

His interest in electrophysiology emerged after he had access to the study published by Scherlag et al. in 1969, demonstrating the feasibility of recording His bundle-electrogram in humans through vascular access.^2^ In the following year, with professors Giovanni Bellotti, João Tranchesi, Radi Macruz and Donaldo Pereira Garcia, he managed to get the first His bundle-electrogram recording at HC-FMUSP and, in 1972, they made the first attempt at the surgical treatment of Wolff-Parkinson-White (WPW).^3^

One day, in 1974, Bellotti contacted Sosa and invited him to study electrophysiology in the WPW Syndrome for his doctoral thesis. They then had to learn to analyze electrophysiological tracings. Sosa was then introduced to this new world with countless challenges.

The first was to talk to Prof. Antonio Paes de Carvalho, Full Professor at Instituto de Biofísica e Fisiologia Carlos Chagas at UFRJ, great scientist and researcher of the electrophysiological mechanisms of cardiac arrhythmias at an experimental level, and internationally recognized.^4^ With him, Sosa went to learn how to obtain and analyze intracardiac electrograms.

After a few days, he did the first electrophysiological study at HC, with Bellotti. Interestingly, the first tracings were rudimentary, with interferences and deflections everywhere. Bellotti would ask: “Sosa, what's the H?” — as there were three deflections, the question remained unanswered. But they learned that records should have less interference to be reliable. Sosa, in his pursuit of perfection, devised ways to achieve high-quality records for the time. (Figure 2)

**Figure 2:** Electrophysiological record taken at HC-FMUSP in 1970, documenting the effect of venous infusion of verapamil on the basic intervals of the conduction system.

Then, patient studies for Bellotti's thesis begin. They would spend the day inducing tachycardias with electrode-induced stimuli, analyzing tracings and interpreting the findings. At the end of each study, they felt deep satisfaction with the findings.

But the biggest surprise occurred on the day of Bellotti's thesis presentation. At the end of the presentation, the examiners praised the innovative study and wrapped up the questions & answers session in about 20 minutes. To date, this has been one of the University's shortest thesis presentations, possibly because the examiners knew little or nothing about the subject.^5^

In 1975, Bellotti was appointed head of the Cardiology ICU, on the 6^th^ floor of HC. At that time, José Antonio Frachini Ramires started to work with him as an assistant of Internal Medicine and Cardiology ICU. “When we met Sosa in the corridors of the Hospital, with electrophysiological sheets in his hands, if he wanted to show us any details, we should be prepared to spend a long time helping him to unfold them, see the records and then fold them back again. Some assistants from the clinic escaped these meetings,” Ramires says.

On that occasion, Cesar Grupi joined the group, helping him to carry out the first electrophysiological studies.^6^^,^^7^ At that time, the studies were carried out under different conditions, such as in patients with acute myocardial infarction, as suggested by Prof. Radi Macruz and evaluation of the electrophysiological effect of antiarrhythmic drugs as some great news…^8^^,^^9^

With the transfer of Cardiology to Instituto do Coração, which had just been opened, Sosa served as head of the valve group and the incipient arrhythmia group. At his request, Bellotti and Prof. Fúlvio Pilleggi created two independent groups: the Arrhythmia Group and the Valvular Heart Disease Group, which remain so until today.

The cardiac electrophysiology laboratory was structured in 1980, becoming a key piece in the development of interventional treatment of various arrhythmias at Incor. Seeking improvement in their procedures, both visited services in the United States, particularly Kenneth Rosen's in Chicago and Mark Josephson's in Philadelphia.

It is worth noting that when they visited these services, they had done 10 WPW surgeries and 10 ventricular tachycardia surgeries, which impressed the local professors, as very few American services performed these procedures at the time.

Notably, Sosa and Miguel changed the paradigm of arrhythmia treatment in Chagas' disease, demonstrating that the recurrent sustained ventricular tachycardias were reentrant circuits related to scarring, and could be reproduced with programmed ventricular stimulation. More importantly, they observed that such circuits originated more frequently in a scar located in the lower, lateral and basal left ventricular wall, as, until then, it was believed that the apical aneurysm, so frequent in patients with Chagas disease, were the focus of such arrhythmias.^10^ (Figure 3)

**Figure 3:** A and B) Intraoperative mapping of a patient with recurrent ventricular tachycardia secondary to Chagas' heart disease in the 1980s. A) Access for endocardial mapping was through the apical aneurysm. Note that point-to-point mapping was performed by bipolar electrodes attached to a device that allowed its direct positioning by the surgeon's finger. B) Electrophysiological records document the endocardial activation sequence of ventricular tachycardia induced in surgery by programmed stimulation.

Throughout the 1980s and 1990s, the surgical treatment program for atrial, supraventricular and ventricular tachyarrhythmias progressed intensely, making Incor a reference center for the surgical treatment of refractory tachyarrhythmia and for the education of electrophysiologists and surgeons, not only from Brazil, but also from Latin America.^10^^–^^15^

In 1982, Gallagher et al.^16^ published the first experience with catheter ablation to induce total atrioventricular block in patients with intractable supraventricular tachyarrhythmias. Once aware of this innovation, Sosa was able to develop connections between defibrillators and conventional catheters, with the help of Adib Jatene.

With Augusto Scalabrini and Silvio Barbosa, they performed the first catheter ablation successfully, using direct electrical discharge (Fulguration) to induce complete atrioventricular block (CHB).^17^ These procedures were performed with conventional electrode catheters, as in the few international services that also began to perform it. It was then that the first structured laboratory of Interventional Electrophysiology was developed in Brazil, as a result of his innovative spirit.

During his administration as director of the Arrhythmia Unit, still in the 1980s, Sosa encouraged the development of the clinical practice of artificial cardiac stimulation with Martino Martinelli as an assistant and Silvana d'Ório and Anísio Pedrosa as contributors, which resulted in the Arrhythmia and Pacemaker Unit.^18^^,^^19^

With the expansion of InCor and an increased number of outpatient care rooms, in the early 90s, Sosa encouraged the creation of a syncope clinic attached to an autonomic evaluation laboratory; a teaching clinic; atrial fibrillation; ventricular tachycardia and genetics, and was helped by new assistants, Denise Hachul and Francisco Darrieux, who jointly developed a university extension program in clinical arrhythmia.^20^^,^^21^

Returning to the 70s, Ramires says that in his master's thesis on the topic of autonomic block in Chagas' disease patients, Sosa inspired the use of beta-blockers in patients with acute myocardial infarction and heart failure. Its benefit was proven through myocardial metabolic assessment and hemodynamic monitoring at the bedside. Since then, the use of beta-blockers was introduced in the therapeutic routine of both pathologies at our institution.^22^^,^^23^

But his great academic interest has always been interventional electrophysiology, on which he worked intensively with his assistant Mauricio Scanavacca. They trained countless electrophysiologists over the years, now responsible for arrhythmia and electrophysiology services not only in Brazil, but also abroad.^24^^–^^31^

In 1984, with fellow cardiologists involved in the treatment of patients with cardiac arrhythmias, particularly Ivan Maia in Rio de Janeiro, Adaberto Lorga in São José do Rio Preto, João Pimenta at Hospital do Servidor Público Estadual and Julio Gizzi at Instituto Dante Pazzanese de Cardiologia, both in São Paulo, founded the Arrhythmia, Electrophysiology and Artificial Cardiac Stimulation Study Group, embryo of what later became the electrophysiology department of SBC and later the Brazilian Society of Cardiac Arrhythmias. (Figure 4 A and B)

**Figure 4:** Two moments of Dr. Sosa participating in the scientific meetings of the specialty. A: Still young, at the first SBC Day of Cardiac Arrhythmias in Rio de Janeiro in 1984 and B: older in the mid-2000s, in one of the congresses of the Department of Cardiac Arrhythmias of SBC.

In the mid-90s, a significant challenge faced by electrophysiology was achieving better results in the ablation of ventricular tachycardia (VT). In our community, this challenge seemed to be greater in patients with Chagas' heart disease.

Noting the low rate of ablation success in these patients, Sosa, Mauricio and the anesthesiologist from João Piccioni's team developed the technique for mapping and epicardial ablation of ventricular tachycardia and demonstrated that the circuits were predominantly epicardial in patients with Chagas' heart disease.^32^^–^^36^ (Figure 5) The group's findings, at that time with the participation of André D'Avila, reached the international scene and the technique started to be used in other types of arrhythmias, in addition to Chagas' disease VT.^31^^,37^

**Figure 5:** Figures from the cover of the Journal of Cardiovascular Electrophysiology of June 1996, promoting the article that described the technique of percutaneous access to the pericardial space. A: Pericardial access by subxiphoid puncture; B: epicardial mapping with catheter; C: records of bipolar electrograms during ventricular tachycardia in a patient with Chagas' disease demonstrating the presence of an epicardial circuit.

Since then, congresses of the American and European arrhythmia and cardiology societies have been a constant stage for presentations of epicardial mapping and ablation, including dedicated sessions, many of which are chaired and moderated by their creators.

After the publication of a study with findings from the new technique, a number of European, Latin American and North American electrophysiologists contacted InCor to learn the technique at the Arrhythmia and Electrophysiology Unit, in order to implement it in their services, such as Cleveland Clinic, Mayo Clinic, University of California, Stanford, Massachusetts General Hospital and several European Reference Centers. This was one of the peaks in the study by “discontented” Sosa, who was always looking for more.

There is no doubt that Sosa created a school with many disciples throughout Brazil, Latin America and other continents. Eduardo Argentino Sosa, Argentine by birth and Brazilian by heart, had a vision focused on the infinite.

Eduardo Sosa will remain forever in the memory of his friends, in the history of Incor and Brazilian Cardiology and his legacy will be immortalized by his disciples.
